# Perioperative reactive oxygen species in infants with biliary atresia: A retrospective observational study

**DOI:** 10.1097/MD.0000000000021332

**Published:** 2020-07-31

**Authors:** Naoki Hashizume, Yoshiaki Tanaka, Kimio Asagiri, Suguru Fukahori, Shinji Ishii, Nobuyuki Saikusa, Motomu Yoshida, Ken Tanikawa, Takahiro Asakawa, Minoru Yagi

**Affiliations:** a Department of Pediatric Surgery, Kurume University School of Medicine; b Division of Medical Safety Management, Kurume University Hospital; c Department of Pediatric Surgery, St Mary's Hospital; d Departments of Pathology, Kurume University School of Medicine, Kurume, Fukuoka, Japan.

**Keywords:** biliary atresia, 8-hydroxy-2’-deoxyguanosine, 8-iso prostagrandinF_2α_, oxidative stress, superoxide dismutase

## Abstract

Biliary atresia (BA) is a devastating cholestatic disorder of infants that presents during the first several months after birth due to an idiopathic obstruction to the bile flow. Without prompt diagnosis, Kasai portoenterostomy, and deliberate follow-ups, the resulting cholestasis leads to progressive hepatic failure. Oxidative stress is an abnormal phenomenon inside cells or tissues caused by a disturbance in the reactive oxygen species (ROS). We aimed to measure perioperative ROS in BA patients.

Data are presented as median (25th, 75th percentiles). We evaluated 15 BA patients (age 55 [48, 69] days) and measured ROS; serum superoxide dismutase (SOD), urinary 8-iso prostaglandin F_2α_ (8-iso-PGF_2α_) and 8-hydroxy-2′-deoxyguanosine (8-OHdG) preoperatively and 30 days later to compare values with serum liver function tests and histologic grades of liver cholestasis. For compared BA patients, 4 normal subjects as control group (age 55 [27, 75] days) measured ROS and serum liver function tests.

In BA patients, the preoperative serum SOD was 6.1 IU/mL (4.7, 7.2), urinary 8-iso-PGF_2α_ was 1969 pg/mg Cre (1697, 2374), and urinary 8-OHdG was 37.1 ng/mg Cre (33.1, 53.7). At the postoperative day 30, the serum SOD was 5.2 IU/mL (4.2, 6.7), urinary 8-iso-PGF_2α_ was 1761 pg/mg Cre (1256, 3036), and urinary 8-OHdG was 42.1 ng/mg Cre (29.65, 72.64). In ROS, there were no significant differences between the 2 periods. In control group, urinary 8-iso-PGF_2α_ was significantly lower than that in preoperative BA patient group. However, other ROS were not significant differences between control group and BA patient group. The concentration of urinary 8-iso-PGF_2α_ was positively correlated with total bilirubin and direct bilirubin levels (preoperatively: *r* = 0.6921, *P* = .0042 and *r* = 0.6639, *P* = .007, postoperatively: *r* = 0.6036, *P* = .0172 and *r* = 0.6464, *P* = .0092, respectively). The preoperative ROS were not correlated with histologic grades of liver cholestasis. Various factors such as liver inflammation, lipid malabsorption, and tissue disorders due to jaundice might affect the antioxidant activity and elevated urinary 8-iso-PGF_2α_. However, at least until 30 days later, urinary 8-OHdG as oxidative DNA damage might persist after the operation whether the cholestasis improved or not.

## 1 Introduction

Biliary atresia (BA) is a devastating cholestatic disorder of infants that presents during the first several months after birth due to an idiopathic obstruction to the bile flow. Without prompt diagnosis, Kasai portoenterostomy (KP), and deliberate follow-ups, the resulting cholestasis leads to progressive conjugated hyperbilirubinemia, cirrhosis, and hepatic failure. The BA patients and end-stage liver disease have no other option but to undergo liver transplantation.

Oxidative stress is an abnormal phenomenon inside cells or tissues caused by a disturbance in the balance between the production of reactive oxygen species (ROS) and both enzymatic and nonenzymatic antioxidants.^[1]^ Excess free radicals can damage fatty tissues and DNA leading to abnormal gene expression, with receptor activity and proliferation disturbances or cell death.^[2]^

**Figure 1:**
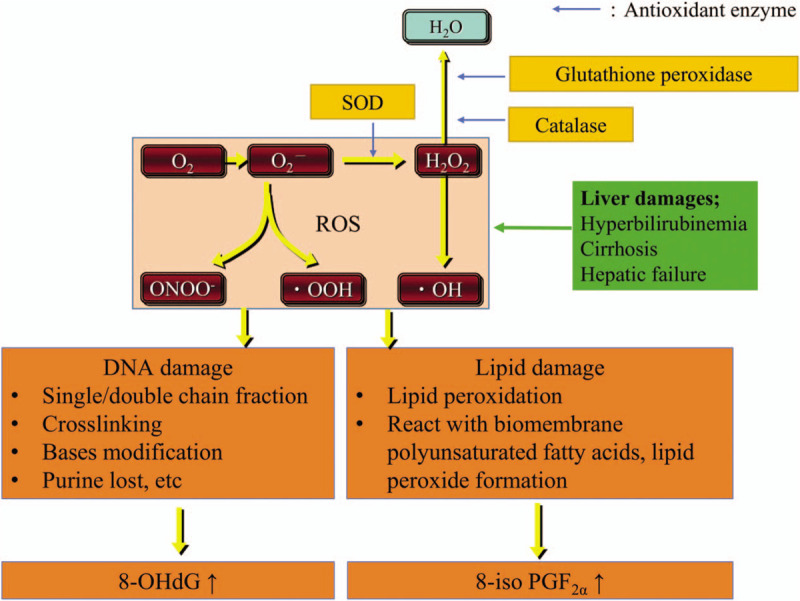
Cellular liver damage associated with reactive oxygen species (ROS) production. Overproduction of reactive oxygen species results in oxidative stress. Antioxidant enzymes catalyze the decomposition of ROS. One antioxidant enzyme is superoxide dismutase (SOD), which is a metalloenzyme capable of scavenging superoxides. 8-Iso-prostaglandine F_2α_ (8-iso-PGF_2α_) is generated by the nonenzymic free radical-initiated peroxidation of arachidonic acid which is present in phospholipid membranes. 8-Iso-PGF_2α_ has been proposed as a valuable biomarker for the assessment of oxidative stress. 8-Hydroxy-2′-deoxyguanosine (8-OHdG) is the product of the oxidation of guanine, an intracellular compound and a component of DNA. 8-OHdG antibody has been widely used to evaluate oxidative DNA damage in animal and human tissues.

Antioxidant enzymes in the cells include superoxide dismutase (SOD), glutathione peroxidase, and catalase (Fig. 1). SOD is a metalloenzyme capable of scavenging superoxide radicals by catalyzing their dismutation to hydrogen peroxide and oxygen.^[1,3]^ This function appears to protect cells from the toxic effects of the endogenously generated superoxide radicals. SOD is a measure of antioxidant activity.

Arachidonic acid is a ubiquitous polyunsaturated fatty acid present in phospholipid membranes in humans. F_2_ isoprostanes are generated by nonenzymatic free radical-initiated peroxidation of arachidonic acid and are frequently used as an accurate measure of oxidative stress during lipid damage.^[4–8]^ One isomer of the F_2_ isoprostane family, 8-iso-prostaglandin F_2α_ (8-iso-PGF_2α_) has been proposed as a valuable biomarker of phospholipid membranes for oxidative stress assessments.^[1,5,7]^ 8-Iso-PGF_2α_ can be detected in plasma, urine, and other biologic fluids.^[4,5,7,8]^

8-Hydroxy-2′-deoxyguanosine (8-OHdG) is a product of the oxidation of guanine, an intracellular component of DNA. 8-OHdG is a relatively stable substance outside the cells produced during DNA repair processes and gets excreted through the blood into urine.^[1]^ Urinary 8-iso-PGF2a and urinary 8-OHdG are measures of ROS (Fig. 1).

In another study, we found that postoperative BA patients and a median age of 5.2 years had significantly increased production of ROS, including higher serum SOD, urine 8-iso-PGF_2α_, and 8-OHdG, than individuals without BA.^[9]^ However, studies on serum and urinary ROS in the pediatric population with liver disease are few,^[9–11]^ and those measuring serum SOD, urinary 8-iso-PGF_2α_, and urinary 8-OHdG levels are even more scarce. Moreover, we know of no reports of the assessment of ROS during the perioperative BA period.

This study aimed to assess the serum and urinary OSAs during the perioperative BA period focusing on serum SOD, urinary 8-iso-PGF_2α_, and urinary 8-OHdG levels.

## 2 Materials and methods

### 2.1 Patients

We enrolled 15 patients (5 boys and 10 girls) with BA and 4 subjects (3 boys and 1 girl) with an umbilical hernia as the control group who were patients from 2006 to 2017 at the Kurume University School of Medicine. In this period, we excluded four patients, 1 with BA operated during day 4, 1 with BA and intracranial hemorrhage due to vitamin K malabsorption, and 2 with sepsis after the operations.

### 2.2 Study design

In 15 BA patients, preoperative sample collections were performed at days 1 or 0 and the postoperative serum sample collections on day 30, defining the day of the operation as day 0. We measured aspartate transaminase (AST), alanine aminotransferase (ALT), total protein (TP), albumin (Alb), alkaline phosphatase (ALP), gamma-glutamyltranspeptidase (GGT), total bilirubin (T-Bil), direct bilirubin (D-Bil), cholinesterase, and total biliary acid levels. In addition, we obtained type IV collagen levels during the preoperative period.

We also measured the following ROS: serum SOD, urinary 8-iso-PGF_2α_, and urinary 8-OHdG. Serum SOD was measured by nitrous transformation (Roche Diagnostics, Tokyo, Japan), urinary 8-iso-PGF_2α_ by an enzymatic immune-assay (8-Isoprostane EIA kit; Cayman Chemical Co, Ann Arbor, MI), and urinary 8-OHdG by an enzymatic immunoassay (ELISA System kit; Institute of Senescence Control of Japan, Shizuoka, Japan). Liver biopsies were performed during the KP procedures, and a pathologist (KT) examined the specimens after fixing them in formalin and staining them with hematoxylin/eosin. Preoperatively, all the BA patients fed breast milk and milk included medium chain triglyceride.

In 4 patients as control group, we measured AST, ALT, TP, Alb, ALP, GGT, T-Bil, D-Bil, serum SOD, urinary 8-iso-PGF_2α_, and urinary 8-OHdG. All patients in control group fed breast milk.

To correlate histologic findings with liver cholestasis disease, we used a published grading system,^[12,13]^ and our preliminary experiment results were based on 4 pathologic parameters including the presence of a portal area bile plug, cholestasis, hepatocyte degeneration, and portal area inflammatory cell infiltration. We used a 12-point histologic scoring system to score the severity of liver cholestasis disease. We classified portal ductal bile plugs into 3 grades: grade 0, bile plug absent; grade 1, bile plugs present without bile lake formation; and grade 2, bile plugs present with bile lake formations. We also classified cholestasis into 3 grades based on the scope of bile pigment deposition and distribution: grade 1, mild cholestasis (pigment deposition within the bile capillary); grade 2, moderate cholestasis (pigment deposition in the small bile duct or hepatocytes); and grade 3, severe cholestasis (diffuse bile pigment deposition in the lobular bile capillary and small bile ductules). We divided hepatocyte degeneration into four grades: grade 1, swollen; grade 2, ballooning degeneration; grade 3, multinuclear hepatocyte formation; and grade 4, necrosis. Additionally, we subclassified portal area inflammatory cell infiltration into 3 categories: stage 1, portal area inflammatory cell infiltration <20%; stage 2, portal area inflammatory cell infiltration <50% without lymphocyte accumulation; and stage 3, portal area inflammatory cell infiltration (including lymphocytes) >50%.

We divided patients into 2 groups: group A (n = 8) comprised patients with T-bil ≥2.0 mg/dL on postoperative day 30 and group B (n = 7) comprised patients with T-bil <2.0 mg/dL on postoperative day 30.

### 2.3 Ethical approval and studies and informed consent

We obtained serum, urinary, and hepatic biopsies after obtaining written informed consents from the patients’ guardians. The ethics committee for clinical research of Kurume University School of Medicine approved this study (approval number 2007-008).

### 2.4 Statistical analysis

Data are presented as median (25th, 75th percentiles). First, the comparison between preoperative BA patients group and control group were performed. Second, the comparison between preoperative and postoperative periods in BA patients was performed. Finally, the comparisons between the 2 subgroups; group A and group B were performed. All comparisons were using the Mann–Whitney *U* and Chi-squared tests. The Spearman rank correlation analysis to assess the association between liver function tests and ROS and to evaluate the correlation between the labeling index and the grades was used. All of the statistical analyses were performed using the JMP Pro 14 software package (SAS, Cary, NC), and *P* values of <.05 were considered statistically significant.

## 3 Results

Table 1 shows the characteristics of BA patients and normal subjects. During the preoperative period, all BA patients showed jaundice and cholestasis liver injury. At the postoperative day 30, AST, T-bil, and D-bil levels were significantly lower than those in the preoperative period in all cases, respectively. There were no significant differences between the other preoperative and postoperative liver function test. There were no significant differences between the preoperative and postoperative ROS.

**Table 1:**
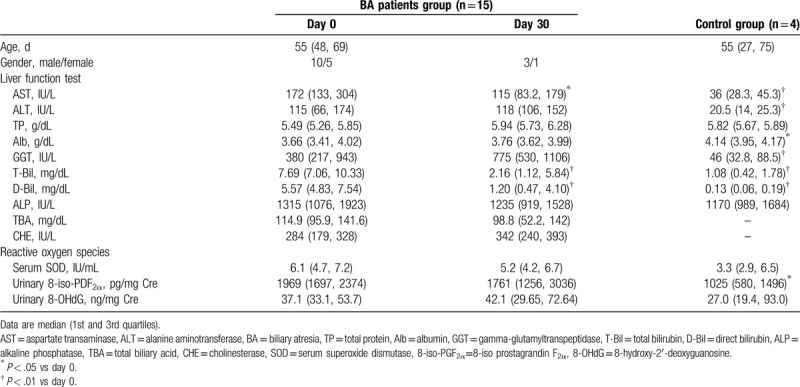
The characteristics of BA patients and control groups.

In age and gender, there was no difference between BA patient and control groups. At the control group, AST, ALT, Alb, GGT, T-bil, and D-bil levels were significantly lower than BA patient group in the preoperative period, respectively. At the control group, urinary 8-iso-PGF_2α_ was significantly lower than at BA patient group. The serum SOD and urinary 8-OHdG values at the control group were marginally lower than those at BA patient group, but there were not significant differences.

**Figure 2:**
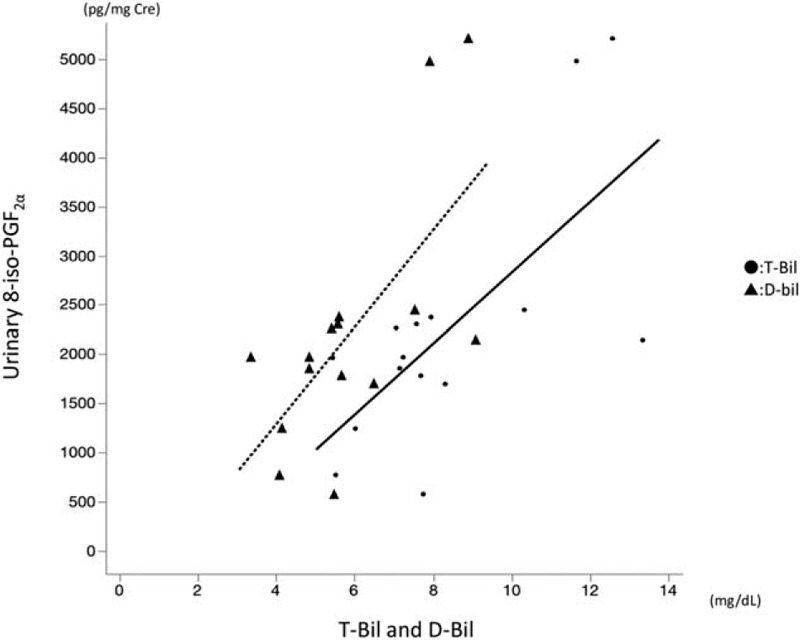
Correlation between preoperative urinary 8-iso-prostaglandine F_2α_ (8-iso-PGF_2α_) and T-bil (solid line), and urinary 8-iso-PGF_2α_ and D-bil (dotted line) (Spearman rank correlation; *r* = 0.6921, *P* = .0042 and *r* = 0.6639, *P* = .007).

**Figure 3:**
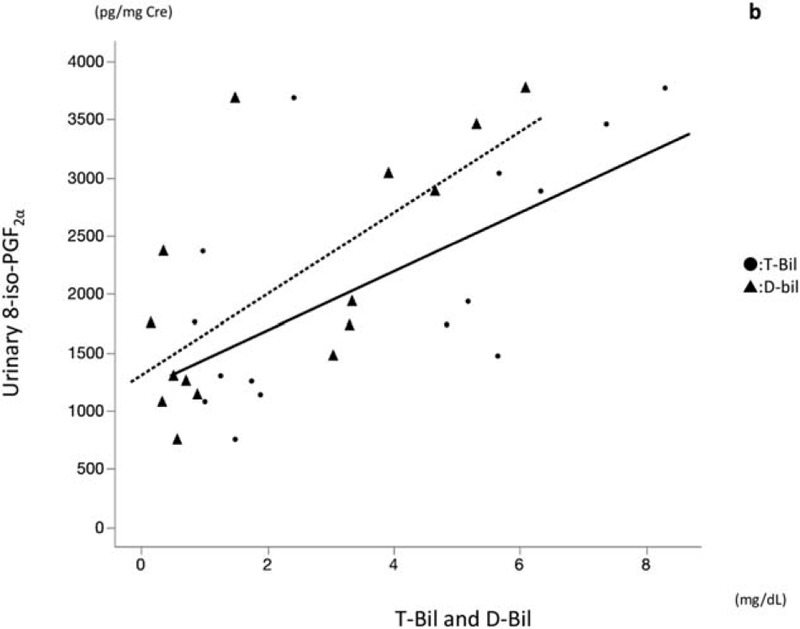
Correlation between postoperative urinary 8-iso-prostaglandine F_2α_ (8-iso-PGF_2α_) and T-bil (solid line), and between urinary 8-iso-PGF_2α_ and D-bil (dotted line) (Spearman rank correlation; *r* = 0.6036, *P* = .0172 and *r* = 0.6464, *P* = .0092).

Preoperatively, the concentration of urinary 8-iso-PGF_2α_ was positively correlated with T-bil and D-bil levels (*r* = 0.6921, *P* = .0042; and *r* = 0.6639, *P* = .007, respectively; Fig. 2). No other preoperative liver function tests were correlated with OSA. At postoperative day 30, the concentration of urinary 8-iso-PGF_2α_ was positively correlated with T-bil and D-bil levels (*r* = 0.6036, *P* = .0172 and *r* = 0.6464, *P* = .0092, respectively; Fig. 3). No other postoperative liver function tests were correlated with ROS.

Table 2 shows the characteristics comparison of BA patients in terms of between group A and group B. There were no significant differences in terms of preoperative liver function test findings between the group A and group B. At postoperative day 30, T-Bil and D-Bil were significantly higher in group A than in the group B. In group A, there were no significant differences between the preoperative and postoperative liver function tests. In group B at the postoperative day 30, the AST, T-bil, and D-bil levels were significantly lower than at the preoperative period, respectively.

**Table 2:**
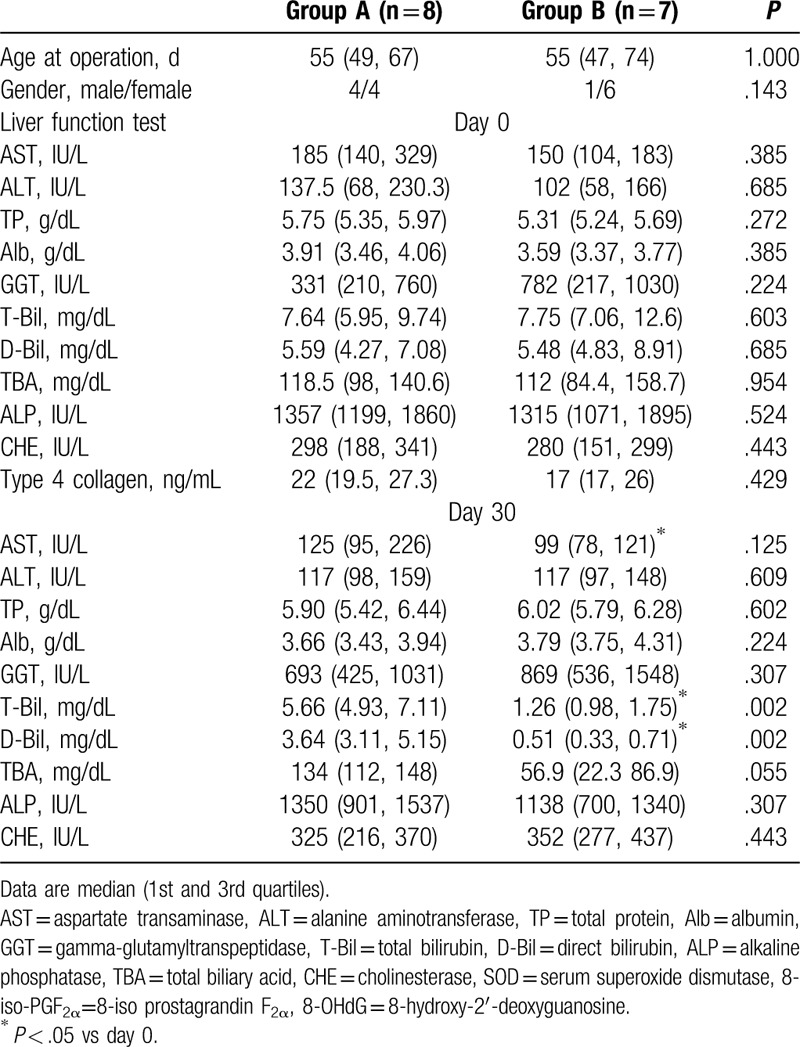
Comparison of the liver function tests between biliary atresia (BA) patients with T-bil ≥ 2.0 mg/dL (group A) and BA patients with T-bil < 2.0 mg/dL (group B) on postoperative day 30.

Table 3 shows the characteristics comparison between group A and group B. There were no significant differences in terms of preoperative ROS between group A and group B. At postoperative day 30, urinary 8-iso-PGF_2α_ was significantly higher in group A than in group B. However, the serum SOD urinary 8-OHdG values were similar in group A and group B. In each group, there were no significant differences between the preoperative and postoperative ROS.

**Table 3:**
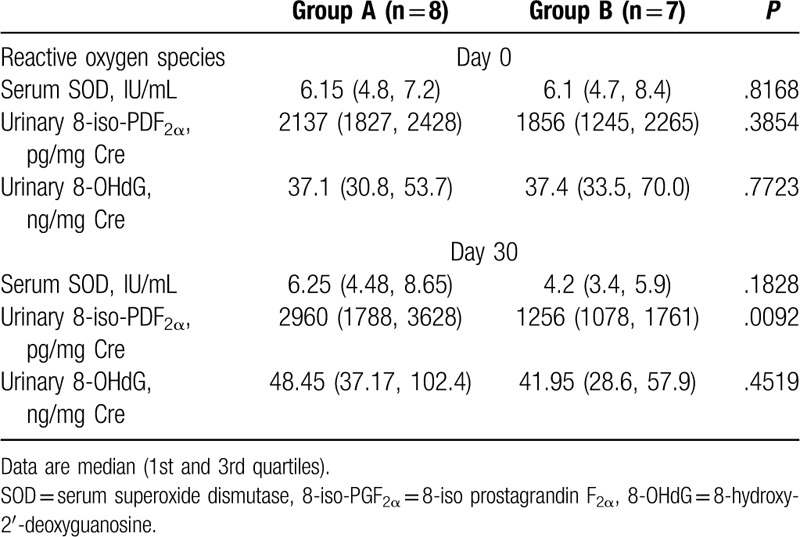
Comparison of the reactive oxygen species between biliary atresia (BA) patients with T-bil ≥ 2.0 mg/dL (group A) and BA patients with T-bil < 2.0 mg/dL (group B) on postoperative day 30.

At the pathologic parameters of BA patients, according to the histologic grades and scores, the bile plugs in portal ductules was classified as 1 (1, 1). All patients had severe cholestasis with diffused bile pigment deposition in the lobular bile capillaries as well as in the small bile duct and were classified the grade of all patients’ cholestasis as grade 3. The hepatocyte degeneration was 3 (2, 4), the portal area inflammatory cell infiltration was 1 (1, 2), and the total scored points were 8 (7, 9.25) respectively. Preoperative OSA did not correlate with the grade or scores of histologic findings.

Table 4 shows the comparison of pathologic parameters between group A and group B. Hepatocyte degeneration was marginally higher in group A than in group B. Portal area inflammatory cell infiltration and the total score was significantly higher in group A than in group B, respectively.

**Table 4:**
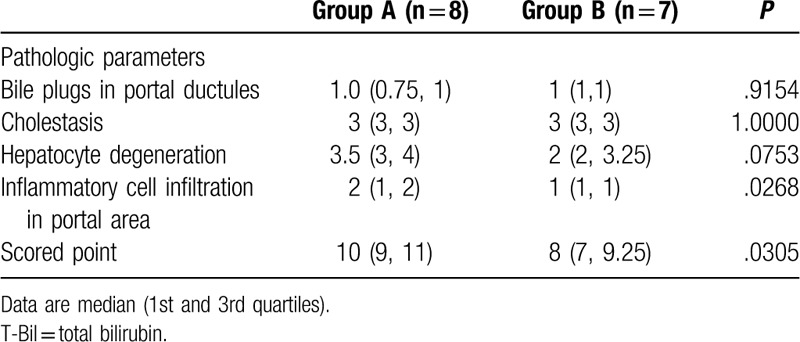
Comparison of the pathologic parameters between biliary atresia (BA) patients with T-bil ≥ 2.0 mg/dL (group A) and BA patients with T-bil < 2.0 mg/dL (group B) on postoperative day 30.

## 4 Discussion

Oxidative stress is known to be involved in the pathogenesis of BA.^[10,14,15]^ SOD in liver tissue is high in infants with cholestatic liver disease such as BA and Alagille syndrome. Moreover, a significant correlation was found between the liver SOD activity and serum bilirubin in BA patients.^[14]^ In patients with cholestatic BA, neonatal hepatitis, and paucity of intrahepatic bile ducts, the levels of SOD, glutathione peroxidase, and catalase in hepatic tissue are significantly increased compared with those in normal liver biopsies.^[10]^ Elevated ROS in the liver reflected hepatocyte damage and metabolized in the liver.^[10]^ Tiao et al reported that the number of 8-OHdG positive hepatocytes was significantly higher in the preoperative liver biopsy of BA patients when the patients had undergone a KP than when they had undergone liver transplantation and choledochal cyst.^[15]^ The early BA stage is associated with a strong inflammatory reaction, with high oxidative DNA included mitochondrial DNA damage (as manifested by higher immunoreactivity, 8-OHdG, and apoptotic activities), and with a decrease in mitochondrial copy numbers. In our study, we found no significant differences between the preoperative and postoperative ROS. The serum SOD and urinary 8-OHdG values at BA patient group were marginally high, but there were not significant differences between control group and BA patient group. Moreover, despite recovery from jaundice, the serum SOD and urinary 8-OHdG levels had not decreased postoperatively at day 30. Our findings suggest that, at least until 30 days later, oxidative DNA included mitochondrial DNA damage persisted after the operation whether the cholestasis improved or not.

Preoperatively, the concentration of urinary 8-iso-PGF_2α_ was positively correlated with both T-bil and D-bil levels. Moreover, postoperatively, urinary 8-iso-PGF_2α_ levels in group A were significantly higher than in group B. Our results suggest that urinary 8-iso-PGF_2α_, reflecting a comprehensive spectrum of lipid peroxidation and antioxidant features, is a significant marker of cholestasis with BA. However, it was suspected that the elevation of urinary 8-iso-PGF_2α_ was not specific to BA and present in all forms of cholestasis. Our findings suggest that production of endogenous steroids and fat-soluble vitamins inhibit the action of the arachidonic cascade.^[16,17]^ In addition, breast milk has important antioxidant properties,^[18,19]^ and improvement of jaundice leads to an increase in milk intake. Improvement in lipid absorption and increase in bile emissions may be affected by the decreased lipid peroxidation.

In our study, preoperative production of ROS did not correlate with histologic grades and scores. In the ligated-bile duct rat model, Baron and Muriel found that although antioxidant treatment inhibits lipid peroxidation and glutathione oxidation, it has no effect on conventional biochemical markers of liver injury or liver histology.^[20]^ The authors speculated that serum and urinary ROS were not correlated with pathologic findings because oxidative stress does not play a causative role in this model of cholestatic liver disease but is instead a consequence of bile salts inducing solubilization of plasma membranes and cell death.^[20]^

We are aware of the limitations of our study. Our analysis was limited due to the small population and the fact that it was based on a single institution. We measured only total SOD activity. Obtaining measurements of subgroups like Mn-SOD (mitochondrial origin) and the Cu-Zu-containing protein in the cytoplasm may provide further information as to whether abnormalities in oxygen free radical metabolism cause or simply reflect tissue injury. Serum and urinary ROS may still be important and useful for appropriate follow-ups of postoperative patients. There were preliminary studies about standard values of serum and urinary ROS for infants.^[5,9,21,22]^ Calculation standard values of serum and urinary ROS for infants is difficult because the standard values change as aging. In terms of the pathologic findings, the liver biopsy specimens were only stained with hematoxylin/eosin and were not evaluated for liver fibrosis. Moreover, extending this study to the include BA outcomes for patients with their native liver in the long term may be a future possibility.

In conclusion, our findings suggest that, in BA patients, urinary 8-iso-PGF_2α_ is high and greatly affected by serum bilirubin. However, serum and urinary ROS were not correlated with pathologic findings. Serum and urinary ROS failed to predict the severity of the preoperative liver injury in BA patients. Various factors such as liver inflammation, lipid malabsorption, and tissue disorders due to jaundice might affect the antioxidant activity and elevated urinary 8-iso-PGF_2α_. However, at least until 30 days later, urinary 8-OHdG as oxidative DNA damage might persist after the operation whether the cholestasis improved or not.

## Acknowledgment

The authors thank enago (https://www.enago.jp) for English language editing.

## Author contributions

**Conceptualization:** Yoshiaki Tanaka.

**Data curation:** Ken Tanikawa.

**Investigation:** Suguru Fukahori, Shinji Ishii, Nobuyuki Saikusa, Motomu Yoshida, Takahiro Asakawa.

**Supervision:** Kimio Asagiri, Takahiro Asakawa.

**Writing – original draft:** Naoki Hashizume.

**Writing – review & editing:** Yoshiaki Tanaka, Minoru Yagi.
